# Mouse Adenovirus Type 1 Early Region 1A Effects on the Blood-Brain Barrier

**DOI:** 10.1128/mSphere.00079-16

**Published:** 2016-04-20

**Authors:** Nagaraja Tirumuru, Carla D. Pretto, Luiza A. Castro Jorge, Katherine R. Spindler

**Affiliations:** Department of Microbiology and Immunology, University of Michigan, Ann Arbor, Michigan, USA; University of Pittsburgh School of Medicine

**Keywords:** adenoviruses, blood-brain barrier, encephalitis, endothelial cells, matrix metalloproteinase

## Abstract

Mouse adenovirus type 1 (MAV-1) infects endothelial cells and disrupts the blood-brain barrier (BBB), causing encephalitis in inbred and outbred mice. Using a virus mutant that does not produce the early region 1A protein E1A, we investigated whether the activity of this known viral transcriptional regulator is needed for BBB disruption and other phenotypes associated with encephalitis. The wild-type (wt) virus and E1A mutant virus caused similar levels of permeability of sodium fluorescein in brains of infected mice. In an *in vitro* assay of BBB integrity, wt and mutant virus caused similar decreases in transendothelial electrical resistance in primary mouse brain endothelial cell monolayers. These results indicate that E1A protein does not contribute to disruption of BBB integrity in animals or cultured cells. Both wt and E1A mutant virus infection of mice led to similar increases in the activity of two matrix metalloproteinases known to correlate with BBB disruption, MMP2 and MMP9, while causing no increase in the steady-state expression of MMP2 or MMP9 mRNA. In contrast, the amount of MMP3 transcripts increased upon infection by both viruses and to a higher level in infections by the mutant virus lacking E1A protein production. There was no difference in the levels of steady-state expression of mRNA for tight junction proteins among mock virus, wt virus, and mutant virus infections. Thus, the MAV-1 E1A protein does not measurably affect BBB integrity in the parameters assayed, although it reduces the amount of MMP3 mRNA steady-state expression induced in brains upon infection.

**IMPORTANCE** Encephalitis can be caused by viruses, and it is potentially life-threatening because of the vital nature of the brain and the lack of treatment options. MAV-1 produces viral encephalitis in its natural host, providing a model for investigating factors involved in development of encephalitis. MAV-1 infection disrupts the BBB and increases activity of matrix metalloproteinases in brains of infected mice. We investigated whether the major transcriptional regulator of adenoviruses, E1A protein, is responsible for any of the specific phenotypes that result from MAV-1 infection. For some of the functions assayed, an E1A mutant virus behaved like wild-type virus. However, expression of mRNA for one matrix metalloproteinase was higher in the virus lacking E1A protein production. This highlights the complex nature of encephalitis and suggests that E1A may have transcriptional effects on host genes important for the development of encephalitis.

## Abstract (precis)

Encephalitis can be caused by viruses, and it is potentially life-threatening because of the vital nature of the brain and the lack of treatment options. MAV-1 produces viral encephalitis in its natural host, providing a model for investigating factors involved in development of encephalitis. MAV-1 infection disrupts the BBB and increases activity of matrix metalloproteinases in brains of infected mice. We investigated whether the major transcriptional regulator of adenoviruses, E1A protein, is responsible for any of the specific phenotypes that result from MAV-1 infection. For some of the functions assayed, an E1A mutant virus behaved like wild-type virus. However, expression of mRNA for one matrix metalloproteinase was higher in the virus lacking E1A protein production. This highlights the complex nature of encephalitis and suggests that E1A may have transcriptional effects on host genes important for the development of encephalitis.

## INTRODUCTION

Encephalitis is an acute inflammation of the brain caused by various agents, including both DNA and RNA viruses of humans (1). Mice infected with mouse adenovirus type 1 (MAV-1; also known as MAdV-1) develop encephalitis in a virus dose- and mouse strain-dependent manner (2–5). MAV-1 causes both acute and persistent infection in mice (6), and it infects cells of the monocyte/macrophage lineage and endothelial cells (2, 7). Infection of brain endothelial cells is thought to lead to encephalitis in MAV-1-infected mice.

Encephalitis is characterized by recruitment of inflammatory cells, cytokine and chemokine secretion, and alteration of tight junction protein levels and localization in endothelial cells, leading to disruption of the blood-brain barrier (BBB) (1, 8–13). The BBB consists of endothelial cells surrounded by pericytes and astrocytes that limit brain entry of inflammatory cells and invading microorganisms (1, 14), thus maintaining central nervous system homeostasis (15). Viral infection and inflammation can lead to acute neuroinflammation and neurodegeneration. MAV-1 infection of primary mouse brain endothelial cells (pmBECs) *ex vivo* reduces tight junction protein levels at the cell surface and overall tight junction protein levels (11). Infection also reduces the barrier function of endothelial cells in culture, manifested as reduced transendothelial electrical resistance (TEER).

It is not known whether specific viral genes contribute to the disruption of the BBB by MAV-1. Our previous work using a mutant MAV-1 that does not produce early region 3 (E3) protein indicated that E3 does not play a role (11). In infections of C57BL/6 mice by the E3 mutant, disruption of the BBB was not markedly different from results seen with infections by wild-type (wt) virus. Also, infections of C57BL/6-derived primary brain endothelial cells by wt virus and the E3 mutant viruses were similar in endothelial cell function and alteration of tight junction protein accumulation on the cell surface. This suggests that the E3 protein does not function in these phenotypes associated with encephalitis.

Another viral gene that might contribute to encephalitis is the MAV-1 early region 1A (E1A) gene. The MAV-1 E1A gene has similarities to the human adenovirus E1A gene, the first viral gene transcribed after the virus enters the cell (16, 17). Human adenovirus E1A is a transcriptional regulator that activates early viral transcription (18, 19). E1A also regulates a number of cellular genes through protein-protein interactions (16). MAV-1 E1A has functional roles similar to those of human adenovirus E1A, for example, binding to the mouse cellular proteins Sur2, retinoblastoma protein (pRb), and pRb-related proteins p107 and p130 (20, 21). MAV-1 strains mutated in E1A are less virulent than wt virus in outbred Swiss mice (6). A mutant virus that eliminates the E1A protein initiation codon and thus produces no detectable E1A protein, *pm*E109, has a 50% lethal dose (LD_50_) that is 5 log units higher than that of the wt virus. The mutant virus is found in the same organs and cell types as the wt virus after intraperitoneal (i.p.) infection, i.e., in the brain, spinal cord, and spleen and in endothelial and mononuclear cells.

Because MAV-1 E1A acts as a regulator of viral and cellular transcription and is important for pathogenesis in mice, we investigated whether it is specifically required for encephalitis-related phenotypes. We infected susceptible SJL mice with wt or E1A mutant virus to assess their virulence in inbred mice. To examine differences between wt and mutant viruses independently of viral load differences (that would occur due to replication differences), we identified infection doses that gave equal viral loads with wt and mutant viruses. Using these doses, we assayed phenotypes that correlate with encephalitis, including BBB permeability to a small-molecule dye and reduction in TEER in primary brain endothelial cells. We measured steady-state transcript levels of tight junction proteins. We also examined enzyme activity and steady-state transcript levels of matrix metalloproteinases (MMPs), which are activated in brains under pathological conditions, including viral encephalitis (1). We found differences in MMP3 transcript levels between wt-infected and *pm*E109 MAV-1-infected mouse brains. In all the other assays, there were no significant differences between the results seen with wt and *pm*E109 MAV-1. Thus, E1A may play a role in encephalitis in MAV-1 infection with respect to MMP3 levels but not in any of the other phenotypes assayed.

## RESULTS

### Lack of E1A affects survival of MAV-1-infected SJL mice.

**FIG 1:**
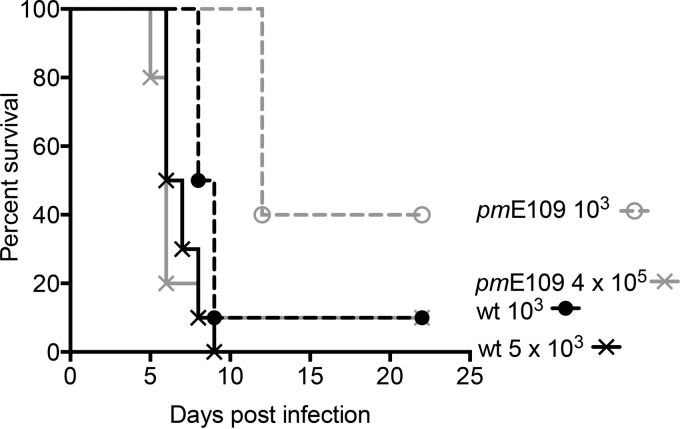
Survival of wt and mutant virus-infected SJL mice. Groups of 10 mice were inoculated intraperitoneally with either wt virus (black symbols and dashed line) or mutant virus (*pm*E109; gray symbols and dashed line) at a dose of 1 × 10^3^ PFU or 5 × 10^3^ PFU (wt; black × symbols) or 4 × 10^5^ PFU (*pm*E109; gray × symbols). The × symbols represent the doses used as indicated in Fig. 2, Fig. 3, Fig. 5, and Fig. 6. Mice were observed twice daily for 22 days. The data were analyzed by the log-rank (Mantel-Cox) test. For the 10^3^ PFU dose, the difference in the survival rates of mice infected with the wt virus versus the *pm*E109 virus was statistically significant (*P* ≤ 0.001).

**FIG 2:**
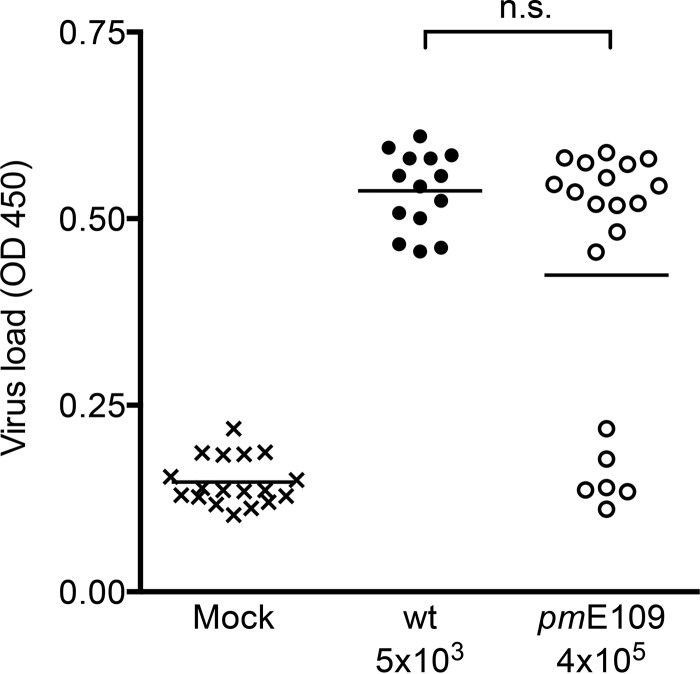
Equal viral loads in wt virus-infected mice and *pm*E109 virus-infected mice. Four-week-old SJL mice were i.p. mock infected (x) or infected with wt (•) or *pm*E109 (o) viruses at the indicated doses. At 6 days postinfection, mice were euthanized, and viral loads in the brain were measured by MAV-1-specific capture ELISA. Positive controls of virus stock, conditioned medium, and PBS were included as controls for the ELISA, and each individual mouse brain was assayed in triplicate. Means are indicated, and the data represent cumulative results of four independent experiments. The differences between the results seen with mock infections and infections using each virus dose shown were statistically significant by one-way analysis of variance (ANOVA) (Kruskal-Wallis analysis with Dunn’s multiple-comparison posttest) (*P* ≤ 0.001). There was no statistically significant difference (n.s.) between wt-infected and mutant-infected mice by this test or Mann-Whitney analysis (*P* = 0.12). OD 450, optical density at 450 nm.

To examine the contribution of MAV-1 E1A to encephalitis, we used a mutant virus, *pm*E109, which was generated by mutating the E1A translation initiation codon (ATG) to TTG, resulting in the absence of detectable E1A protein (21). The *pm*E109 virus is less virulent than the wt virus in outbred Swiss mice and inbred SJL mice (4, 6, 22). Intraperitoneal (i.p.) infection of susceptible SJL mice with equal doses (5 × 10^3^ or 1 × 10^4^ PFU) of wt and *pm*E109 viruses resulted in brain viral loads nearly 2.5 log units higher for wt virus (data not shown), similarly to results seen at a 1 × 10^6^ PFU dose of both viruses (22). To examine how E1A affects survival in SJL mice, we infected groups of 10 mice with wt or *pm*E109 viruses at several doses. Mice infected with the mutant virus survived better at a 10^3^ PFU dose than mice infected with the wt virus at 10^3^ PFU (Fig. 1); similar results were seen at a 10^4^ PFU dose (data not shown). The difference in survival rates of mice infected with wt virus compared to *pm*E109 virus at both doses was statistically significant (*P* ≤ 0.001 and *P* ≤ 0.0001, for 10^3^ and 10^4^ PFU doses, respectively). These results are consistent with previous 50% lethal dose (LD_50_) measurements of the *pm*E109 mutant in susceptible outbred mice, in which the mutant virus LD_50_ was 3 to 5 log units higher than that of wt virus (6). Figure 1 also shows survival experiments with doses of virus that gave equivalent brain viral loads (see Fig. 2 below), i.e., 5 × 10^3^ (wt) and 4 × 10^5^ (*pm*E109).

To examine brain phenotypes relating to encephalitis, we wanted to compare virus infections in which brain viral loads were similar. This would enable us to compare phenotypes due to the E1A mutation rather than phenotypes due just to replication differences that would lead to higher viral loads in wt- versus *pm*E109-infected mice. To identify doses of wt and mutant virus that give equivalent viral loads in infected brains, mice were subjected to mock infection or infected with wt or *pm*E109 virus at a variety of doses (data not shown and Fig. 2). We infected groups of ~5 mice in four independent experiments and pooled the results. Mice were euthanized 6 days postinfection (dpi), and brain viral loads were determined by MAV-1-specific capture enzyme-linked immunosorbent assay (ELISA) (23). Mice infected with 4 × 10^5^ PFU of mutant virus had brain viral loads equivalent to those seen with the mice infected with 5 × 10^3^ PFU of wt virus. That is, the mutant virus dose required to achieve brain viral loads equal to wt MAV-1 loads was nearly 2 log units higher than the wt dose. There was no statistically significant difference between brain viral loads of wt and *pm*E109 virus-infected mice infected at the 5 × 10^3^ and 4 × 10^5^ PFU doses, respectively. Six of the mice in the *pm*E109 group (and wt-infected mice infected at other doses; data not shown) had viral loads similar to those of mock-infected mice at 6 dpi. We sometimes see mice with low viral loads in MAV-1 infections, and we attribute it to biological variability in the kinetics of infection. This may be seen more frequently with the mutant virus because its kinetics are slower (e.g., compare time-to-death data for the 10^3^ PFU doses in Fig. 1). That is, at 6 days postinfection, the mutant virus-infected mice with low viral loads perhaps had not yet progressed to a point where virus replicated to measurable levels. In subsequent experiments of this study, we infected mice with wt virus at 5 × 10^3^ PFU or mutant virus at 4 × 10^5^ PFU doses and assayed additional parameters only in mice that showed viral loads higher than baseline (mock infection) levels.

### Lack of E1A does not alter MAV-1 effects on BBB permeability.

**FIG 3:**
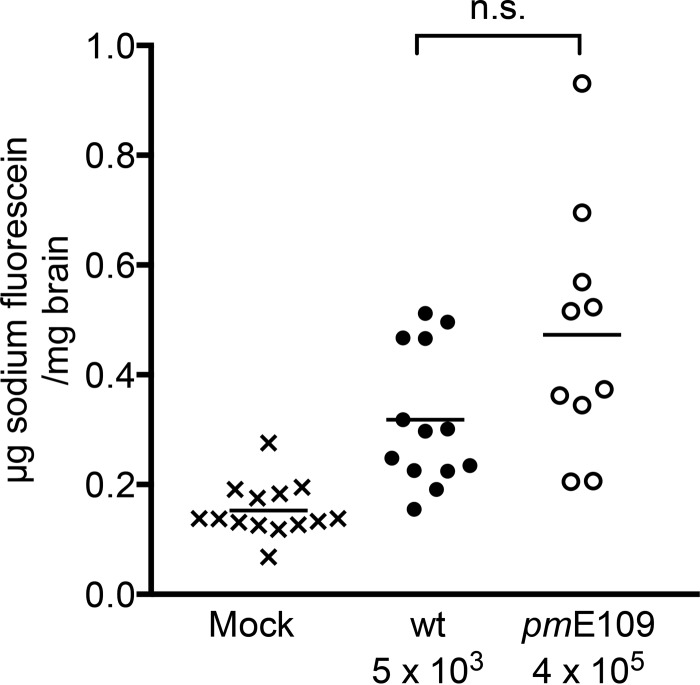
Permeability of sodium fluorescein in wt or *pm*E109 virus-infected mice. SJL mice were subjected to mock infection (x) or were infected with wt (•) or *pm*E109 (o) virus at the indicated doses (which gave equal viral loads; data not shown). At 5 or 6 days postinfection, mice were injected with 10% sodium fluorescein for 10 min and euthanized. Mean levels of sodium fluorescein in the brains are indicated, and the data represent the cumulative results of two independent experiments. By one-way ANOVA (Kruskal-Wallis analysis with Dunn’s multiple-comparison posttest), the differences between the results of mock infections and infections with each virus were statistically significant (mock versus wt, *P* ≤ 0.01; mock versus *pm*E109, *P* ≤ 0.0001), but there was no significant difference between the results seen with the two virus strains. Also, by Mann-Whitney analysis, there was no significant difference between the results seen with the two virus strains (*P* = 0.06).

We analyzed the role of the E1A gene in brain phenotypes related to encephalitis and BBB disruption, using mice that had been mock infected or infected with wt or *pm*E109 virus at doses that yielded equal brain viral loads. At 10 min before euthanasia, the mice were injected with sodium fluorescein, a small-molecule dye that does not penetrate the brain unless there is damage to the BBB. Serum was collected, mice were perfused with phosphate-buffered saline (PBS), and brains were collected for the analysis of sodium fluorescein uptake. As shown in Fig. 3, there was higher sodium fluorescein uptake in wt and *pmE*109 virus-infected mice than in mock-infected mice, and this was statistically significant. There was no significant difference in levels of sodium fluorescein uptake among groups of mice infected with wt or mutant virus. Brain viral loads were confirmed to be equivalent among the wt and mutant virus-infected mice (data not shown).

**FIG 4:**
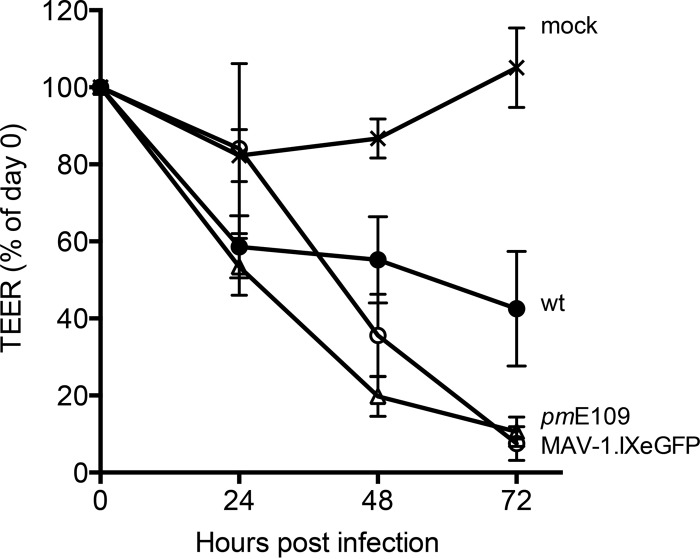
Barrier properties in pmBECs infected with wt or *pm*E109 mutant virus. pmBECs from SJL mouse brains were grown to confluence on transwell plates with astrocyte-conditioned medium in the bottom chamber and complete pmBEC growth medium plus 500 ng/ml hydrocortisone in the upper chamber to promote tight junction formation. Cells were infected with wt (•), *pm*E109 (o), or MAV-1.IXeGFP (Δ) virus at an MOI of 5 or were mock infected (x). Transendothelial electrical resistance (TEER) was measured at 24-h intervals. A blank well with no cells was measured throughout the experiment, and the corresponding value was subtracted as the background. Results are presented as percentages of TEER activity on day 0 for each condition. Results shown are from two independent pmBEC preparations, and each point represents the average of results from 3 to 4 wells. Error bars are standard errors of the means. By one-way ANOVA (Kruskal-Wallis analysis with Dunn’s multiple-comparison posttest), the virus-infected samples were not significantly different from one another at any time point. By unpaired *t* test, with Welch’s correction, the results from all virus-infected samples were significantly different from those seen with mock-infected samples at 72 h postinfection (*P* ≤ 0.05).

Infection of pmBECs with MAV-1 reduces the barrier resistance of brain endothelial cells *ex vivo* (5, 11), as measured by a reduction in TEER of pmBECs grown in transwell plates. To determine whether E1A is needed to cause the decrease in TEER observed after infection, we infected SJL pmBECs with wt and mutant MAV-1. After pmBECs reached maximum TEER, we either subjected the mice to mock infection or infected them with wt MAV-1, *pm*E109 mutant virus, or a green fluorescent protein (GFP)-expressing (but otherwise wt) MAV-1 strain, MAV-1.IXeGFP, at a multiplicity of infection (MOI) of 5. We measured TEER at 0, 24, 48, and 72 h postinfection (hpi). At 24 hpi, cells infected with each of the three viruses had decreased TEER readings that were nearly 50% of those seen at 0 hpi or of those seen with mock-infected cells at 0 or 24 hpi (Fig. 4). At 72 hpi, cells infected with wt, *pm*E109, or MAV-1.IXeGFP virus had dramatic decreases in TEER to nearly background levels compared to mock-infected cells. Wild-type virus-infected cells showed a slight lag in their TEER decrease compared to *pm*E109 mutant virus-infected or MAV-1.IXeGFP virus-infected cells. The kinetics and amount of decrease in TEER are consistent with previous results for wt virus infection (5, 11). All the viral samples had significantly reduced TEER at 72 hpi relative to mock-infected pmBECs; there was no statistically significant difference in the TEER values between wt viruses and the E1A mutant. These data indicate that E1A function does not directly contribute to viral disruption of barrier properties of pmBECs.

### MMP activity is increased in virus-infected mouse brains.

**FIG 5:**
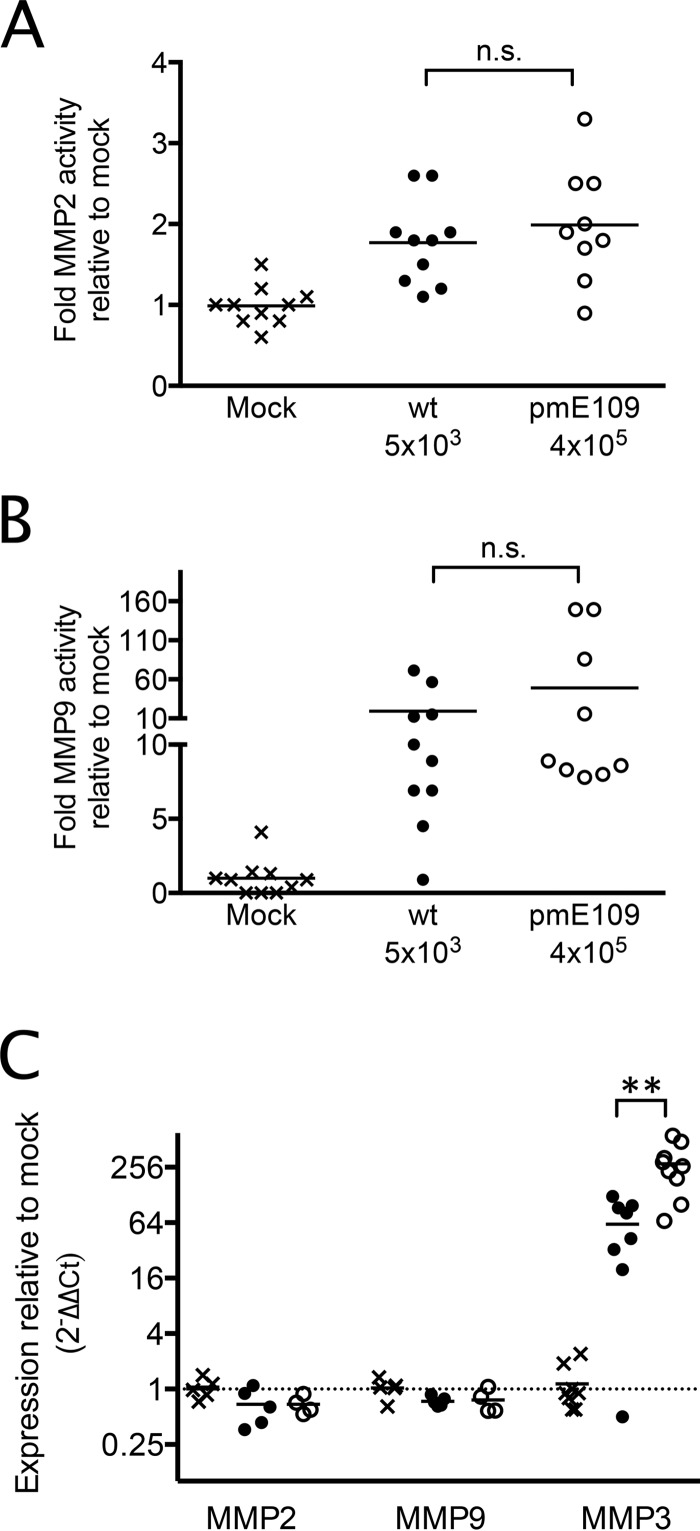
MMP enzyme activity and mRNA levels in wt and mutant virus-infected mouse brains. Brains from mice that were mock infected (x) or infected with wt (•) or *pm*E109 (o) virus and that had equal brain viral loads (data not shown) were analyzed for MMP activity and mRNA levels. (A and B) Brains were homogenized and analyzed for MMP2 (A) or MMP9 (B) activity by gelatin zymography. The data are presented as fold activity in infected brains relative to mock-infected brains for each enzyme. For MMP2 activity, differences in means between wt virus-infected and mock-infected brain results and between pmE109 virus-infected and mock-infected brain results were significant (*P* ≤ 0.01 and *P* ≤ 0.001, respectively). For MMP9 activity, differences in means between mock and *pm*E109 infection results were significant (*P* ≤ 0.05). There was no statistically significant difference (n.s.) between means of activity for wt and *pm*E109 virus infections for either MMP. (C) MMP2, MMP3, and MMP9 steady-state mRNA levels in mouse brains were determined by TaqMan RT-qPCR. mRNA levels were normalized to GAPDH expression for each sample; normalized values for the infected samples were then normalized to those for the means of the results determined for mock-infected samples and are represented as fold change in RNA level relative to mock infection results (indicated by the horizontal dashed line). Sample data represent the cumulative results of two independent experiments. Means are indicated, and differences between the results of mock infections and infections with each virus for MMP3 mRNA levels were statistically significant by one-way ANOVA (Kruskal-Wallis analysis with Dunn’s multiple-comparison posttest) (*P* ≤ 0.0001). The difference between wt and *pm*E109 means was also statistically significant by the same test (**, *P* ≤ 0.01).

In the brain, activity of MMP2, MMP3, and MMP9 is induced under pathological conditions, including viral infection (1). MMP3 and MMP9 can be transcriptionally induced by cytokines and are involved in inflammatory responses (24, 25). MMP2 is constitutively present in zymogen form throughout the brain and activated upon host response to injury. Activation of MMPs occurs via cleavage by other MMPs or proteases or by exposure to free radicals; in particular, MMP9 can be proteolytically activated by MMP3 (24, 26). MMPs can cause BBB disruption by degradation of the basement membrane and extracellular matrix and by cleavage of tight junction proteins (27–30). A number of viruses that disrupt the BBB have increased MMP activity or expression upon infection (reviewed in reference 1). We examined whether mice infected with wt MAV-1 or *pm*E109 mutant virus differed in their MMP2 and MMP9 enzyme activity or steady-state mRNA levels in infected brains. Using mice infected with wt and *pm*E109 that gave equal brain viral loads, mouse brain homogenates were assayed for MMP activity by gelatin zymography. As shown in Fig. 5A and B, MMP2 activity and MMP9 activity were increased in wt and mutant virus-infected brains compared to mock-infected brains. However, there was no statistically significant difference in MMP activity between wt MAV-1 and mutant virus-infected brains.

We also analyzed MMP mRNA levels in the brains. As shown in Fig. 5C, there were no differences in MMP2 and MMP9 mRNA levels among mock-infected, wt MAV-1-infected, or mutant *pm*E109-infected brains. These data suggest that the increased enzyme activity (Fig. 5A and B) was not due to increased MMP2 and MMP9 mRNA levels but rather to a posttranscriptional event such as MMP activation. Further, the data suggest that absence of the E1A protein does not alter MMP2 or MMP9 activity or mRNA levels during infection.

Levels of MMP3 mRNAs were low in mock-infected brains, giving high or undetermined threshold cycle (*C_T_*) values in the quantitative PCR (qPCR) analysis. MMP3 mRNAs were abundantly expressed in both wt MAV-1-infected and *pm*E109 virus-infected brains (Fig. 5C), and there was a higher level of MMP3 expression in the *pm*E109-infected brains. Differences among all three groups were statistically significant. We are not able to measure MMP3 enzyme activity because the signal-to-noise ratio for casein zymography is very low in our hands, but given the known ability of MMP3 to activate MMP9 (24, 26), the high level of MMP3 mRNA levels in infected brains may explain the higher MMP9 enzyme activity in infection shown in Fig. 5B.

### Effect of E1A on tight junction protein mRNA expression.

**FIG 6:**
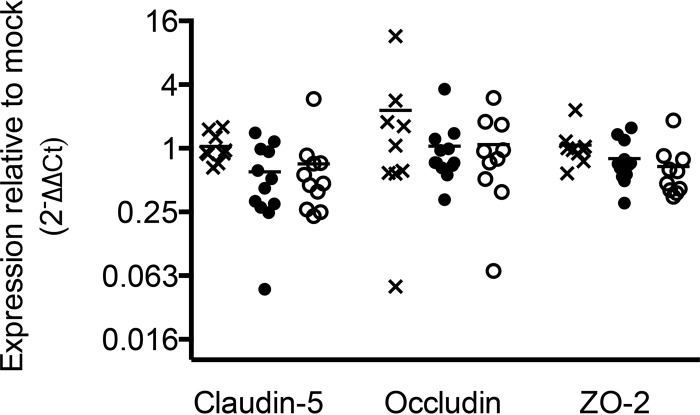
Tight junction protein steady-state mRNA levels. Steady-state mRNA levels for the indicated tight junction proteins in brains from mice that were mock infected (x) or infected with wt (•) or *pm*E109 (o) viruses were determined by RT-qPCR. mRNA levels were normalized to GAPDH RNA levels in each sample; values for the infected samples were normalized to those for the mock-infected sample and are represented as fold change relative to mock infection results. Means are indicated; *n* = 9 to 12 per group. There were no statistically significant differences between mock infection and wt and *pm*E109 virus infection results for any tight junction transcripts.

Tight junction proteins are important for the integrity of the BBB (reviewed in reference 1). Steady-state levels of mRNAs for tight junction proteins claudin-5, occludin, and zona occludens 2 (ZO-2) are lower in wt and E3 mutant virus-infected pmBECs than in mock-infected cells (11). To determine whether E1A modulates steady-state levels of tight junction protein mRNAs in whole brains, we assayed tight junction protein mRNA levels by reverse transcriptase qPCR (RT-qPCR) from brains of mice infected by wt or *pm*E109 virus that gave equal viral loads. As shown in Fig. 6, there was no difference between virus-infected and mock-infected brains for claudin-5, occludin, or ZO-2 mRNA steady-state levels, nor was there a difference between the wt and mutant virus-infected brains.

## DISCUSSION

We examined whether the transcription-modulating E1A gene of MAV-1 contributes to encephalitis in susceptible SJL mice. Using a mutant virus, *pm*E109, which does not produce E1A protein, we demonstrated that E1A is not required for most of the encephalitis phenotypes that we assayed. However, there were higher MMP3 transcript levels in pmE109-infected mouse brains.

The *pm*E109 virus had reduced virulence compared to the wt virus in the inbred MAV-1-susceptible SJL mice. This was consistent with our earlier findings in outbred susceptible mice and SJL mice (4, 6, 22). Similarly to our results reported here, we have shown previously that intraperitoneal infection of inbred or outbred susceptible mice with equal doses of wt and *pm*E109 virus (100 PFU for inbred mice, 1 PFU for outbred) yields higher brain viral loads for the wt-infected mice (6, 22). Differences in lung viral loads for mice infected intranasally with wt and *pm*E109 result in differences in chemokine responses, whereas when viral loads are equivalent, chemokine levels are also equivalent (31). This is likely due to a virus replication defect in the lungs in the absence of MAV-1 E1A. Therefore, to make comparisons of encephalitis phenotypes between mice infected with wt and E1A mutant viruses independently of potential viral replication differences *per se*, we determined doses for each virus that would give equivalent brain viral loads. We found that 5 × 10^3^ PFU of wt virus gave brain viral loads equivalent to those seen with 4 × 10^5^ PFU of *pm*E109 and used those doses throughout this work.

MAV-1 infection causes disruption of the BBB, which can be measured by permeability with respect to small-molecule dyes. Infection of susceptible mice with MAV-1 results in uptake of sodium fluorescein and Evans blue dyes (5, 11). Previous experiments demonstrated no difference in permeability with respect to either dye for C56BL6 mice infected with wt or E3 null mutant virus (11). Results here were similar for experiments investigating E1A contributions to permeability induced by MAV-1 infection. When we compared permeability to sodium fluorescein in susceptible SJL mice using *pm*E109, which does not produce the E1A protein, there was no difference from the permeability in wt virus infections. We also assayed for barrier integrity by using pmBECs from SJL mice, infecting with wt or E1A mutant virus, and measuring TEER. Infections by both wt and E1A mutant viruses caused a substantial drop in TEER by 3 days p.i. Similar results were seen for TEER changes after infection of C57BL6 pmBECs by wt virus and an E3 null mutant virus; i.e., both the wt and mutant viruses caused a drop in TEER. Taken together, these results suggest that neither E1A nor E3 protein function is directly required for the barrier integrity in mouse infections or cultured cells.

MMPs mediate tight junction changes that lead to BBB dysfunction (32, 33) through their proteolytic activity for extracellular matrix and cleavage of tight junction proteins (reviewed in reference 1). Increased activity of MMPs is seen in *in vitro* and *in vivo* infections by viruses that cause encephalitis, including HIV-1 (34–36), flaviviruses (37–39), and herpesviruses (40). MMPs are enzymes that are synthesized as zymogens (proenzymes). Net activity is regulated at the levels of gene expression, activation of proenzymes (by other MMPs or reactive oxygen species), inactivation by tissue inhibitors of MMPs (TIMPs), and cellular compartmentalization (reviewed in reference 1). We examined MMP2 and MMP9 activity here by zymography analysis of whole brains and characterized one of the contributing regulatory mechanisms, expression of mRNA. We found that MAV-1 infection increased MMP2 and MMP9 activity compared to mock infections. This was true for both the wt and *pm*E109 mutant viruses; however, the levels of MMP activity were not significantly different between the two. When we examined mRNA levels for the MMP2 and MMP9 mRNAs in virus-infected brains, they did not differ significantly from mock-infected brain levels. This strongly suggests that the increase of MMP activity is due to activation of proenzymes present in the brain cells, inactivation of TIMPs, or a change in compartmentalization of MMP proenzymes. Our experiments do not distinguish among these possibilities. However, we did observe an increase in MMP3 mRNAs in infection by both wt and the *pm*E109 mutant viruses. MMP3 can activate MMP9 (24, 26), and it is possible that the increase in MMP3 mRNA led to increased MMP3 protein levels and contributed to the increase in MMP9 enzyme activity that we observed upon viral infection. The E1A mutant infection resulted in more MMP3 mRNA in infected mouse brains than did the wt virus infection, and the difference was statistically significant. We are uncertain of the biological significance of this finding.

We found no differences in steady-state levels of tight junction protein mRNAs in whole brains among mock-infected, wt virus-infected, or E1A mutant virus-infected mice. This could suggest that E1A does not modulate transcription of these genes. However, assay of whole brains, where the majority of cells are not infected by MAV-1 (7, 41), may mask any effects of E1A on the subpopulation of brain cells that are infected. When pmBECs are infected *ex vivo*, there is a lower level of tight junction protein steady-state mRNA expression mediated by wt MAV-1 (11); it is possible that E1A modulates this effect, but we have not tested it.

In summary, we examined whether MAV-1 E1A contributes to pathogenesis by altering aspects of BBB integrity. We saw no difference in sodium fluorescein uptake or MMP2 and MMP9 activity between mouse brains infected with viruses with or without the MAV-1 E1A protein. This suggests that E1A does not play a direct role in disruption of the BBB during MAV-1 infection. Consistent with this, we also saw no difference in the abilities of the viruses to disrupt an *in vitro* endothelial monolayer, as measured by TEER. It remains possible that E1A has an effect on other aspects of infection in mouse brains that do not affect BBB permeability, such as modulation of host genes that contribute to inflammation or viral replication. It is also formally possible that an E1A function mediating BBB disruption might be masked by another viral gene with a redundant function. Our data are consistent with E1A not being a primary mediator of brain pathology measured by the assays here.

## MATERIALS AND METHODS

### Cells.

Cell lines were cultured in Dulbecco’s modified Eagle’s medium (DMEM) (Gibco Life Technologies) (pH 7.2) containing 1% penicillin and streptomycin (Gibco). Media for NIH 3T6 mouse fibroblast and CMT-93 mouse rectal epithelial cells contained 5% heat-inactivated calf serum and 10% fetal bovine serum, respectively.

Primary mouse brain endothelial cells (pmBECs) were isolated using methods similar to methods previously described (11). For each preparation, we used 30 mouse brains from 3-week-old male SJL/J mice, isolated cortexes, and disrupted major blood vessels based on visual inspection. We have not noted sex differences in MAV-1 pathogenesis in mice, and our previous studies using *pm*E109 were done in male mice (6, 22). The brains were minced and mechanically homogenized in Hanks’ balanced salt solution (HBSS; Gibco) using a Dounce homogenizer. Cells were pelleted (205 × *g*, 5 min at 4°C) and resuspended in 18% dextran (USB Products). The suspension was centrifuged (9,000 rpm, 10 min, 4°C) in a Sorvall SA-600 rotor. Myelin and liquid supernatant were aspirated, and erythrocytes and microvessels were resuspended in HBSS. The suspension was layered onto a precentrifuged gradient (prepared by centrifuging 46.5% Percoll at 12,500 rpm for 1 h at 4°C in a Sorvall ultracentrifuge using an SW41 rotor) and then centrifuged (2,800 rpm, 10 min at 4°C) in a Jouan benchtop centrifuge. Microvessels formed a distinct red band in the density gradient and were carefully transferred to a 50-ml tube and washed by adding HBSS to make a volume of 50 ml and centrifuging (800 × *g*, 5 min at 4°C) again to form a pellet. The pellet was resuspended in 10 ml of 1 mg/ml collagenase/dispase (Roche Diagnostics) solution and incubated at 37°C for 20 to 40 min. The enzyme solution was then inactivated with the addition of 2 to 3 volumes of HBSS. Microvessels were centrifuged (800 × *g*, 5 min at 4°C). Supernatant was aspirated, and the pelleted material was resuspended in pmBEC growth medium, which consisted of DMEM (Gibco) (pH 7.2) containing 10% fetal bovine serum, 10% newborn calf serum, 2 ml of 10 mg/ml endothelial cell growth supplement (BD Biosciences), 0.1 mg/ml heparin (Sigma), 2 mM glutamine, 1% penicillin and streptomycin (Gibco), 1% antimycotic/antibiotic (Gibco), 1% nonessential amino acids (Sigma), and 20 mM HEPES (Sigma). The cells were plated onto collagen IV-coated plates (BD Biosciences). Puromycin (4 µg/ml) was added 24 h after isolation for 48 h to inhibit growth of fibroblasts and pericytes. For passage, cells were dissociated from the plate using Accutase (EMD Millipore Corporation).

### Mice.

All animal work complied with relevant federal and University of Michigan policies. Mice were housed in microisolator cages and had food and water *ad libitum*. SJL/J male mice (3 to 4 weeks old) were obtained from Jackson Laboratory and used for infection and isolation of primary brain endothelial cells. Mice were infected via i.p. injection in volumes of 100 µl, with virus doses of 10^3^ to 4 × 10^5^ PFU. Virus was diluted in 10-fold serial dilutions in endotoxin-free Dulbecco’s phosphate-buffered saline (DPBS; Lonza). Mice injected with conditioned media similarly diluted in DPBS were considered mock infected. Conditioned medium was obtained from uninfected cells. Mice were monitored at least twice daily for signs of disease and were euthanized by CO_2_ asphyxiation if moribund or at the indicated time points. Organs were harvested, snap frozen on dry ice, and stored at −70°C until further processing.

### Viruses.

Wild-type MAV-1 and the green fluorescent protein (GFP)-expressing virus (see below) were grown and passaged in NIH 3T6 fibroblasts, and titers of viral stocks were determined by plaque assay on 3T6 cells as previously described (42). In mutant virus *pm*E109, the E1A translation initiation codon is mutated to TTG, resulting in the absence of detectable E1A protein (21). The mutant virus was grown and titrated on the E1A-complementing 3T6-based 37.1 cell line as previously described (21).

### GFP-expressing virus construction.

We constructed MAV-1 expressing enhanced GFP (eGFP) fused at the C terminus of the minor capsid protein pIX. We first amplified the gene for MAV-1 pIX from pMXD (17) with a 5′ primer (BamLpIX; 5′GCAAGGATCCTTTCTAACATATGAAAGTG3′) that added a BamHI site 5′ of the NdeI site at nucleotide (nt) 2014 (MAV-1 sequence; NC_000942) and a 3′ primer (ClaH3RpIX; 5′ACGAAAGCTTATCGATATCACTTTCTTCCCCGTT3′) that added ClaI and HindIII sites just before the pIX stop codon, such that the ClaI site followed the last amino acid codon and facilitated the subsequent cloning of eGFP in frame with pIX. This PCR product was digested with NdeI and HindIII and ligated into NdeI- and HindIII-digested pUC8 to create plasmid pUC8pIX. pShuttleIX-EGFP (43), a kind gift of David Curiel (Washington University), was used to amplify the eGFP gene, using a 5′ primer (LR1ClaFLAGGFP; 5′GGAGCAGAATTCATCGATTCTGCCGATTATAAGGATGAC3′) that inserted an EcoRI site and a ClaI site upstream of FLAG-tagged eGFP and a 3′ primer, RSphSal (5′AGTCGTCGACGCATGCATCTTTATTTGGGATCATAACTTGTACAGCTCGTCCAT3′), which includes the sequence from nt 3175 to nt 3195 (SphI site) of MAV-1 followed by a 3′ SalI site. This eGFP product was digested with ClaI and SalI and inserted into ClaI- and SalI-digested pUC8pIX. The resulting plasmid, pUCpIXFGFP, was digested with NdeI and SphI and the smaller fragment inserted into similarly digested pMXD, effectively replacing the wt pIX sequence in pMXD with the pIX-eGFP fusion. This construct, pMXD-FGFPpIX, was verified by DNA sequencing.

We introduced pIX-eGFP into the MAV-1 genome in the region of the pIX gene by bacterial artificial chromosome (BAC) recombineering using GalK selection (44). We amplified the GalK gene from pGalK (44) with a forward primer (MAVL3101; 5′GCACAAGGAGAGGAAGAGGAGGAAGAGGAGGACGGAGCTGAAGACATTGAGCCTGTTGACAATTAATCATCGGCA3′) containing 51 nt of MAV-1 sequence (nt 3101 to 3151) followed by 24 nt of GalK cassette sequence and a reverse primer (MAVL3404; 5′CTGGATCATCTATAACACCTCCCCCGAACATGAATGTATGCAATGGATGTACTCAGCACTGTCCTGCTCCTT3′) containing 52 nt of MAV-1 sequence (nt 3455 to 3404) followed by 20 nt of GalK cassette sequence. The amplified mixture was digested with DpnI to remove methylated plasmid template, and the PCR product was purified. This GalK PCR fragment with 50 bp of MAV-1 sequence flanking each end was introduced by recombineering into *Escherichia coli* SW102 containing a bacmid that has the entire wt MAV-1 sequence, pKBS2.MAV‑1wt (colony16), which was a kind gift of Silvio Hemmi (University of Zürich). Recombineering protocol 3 (http://ncifrederick.cancer.gov/research/brb/protocol.aspx) was used for this and subsequent recombineering steps. Gal-positive (Gal^+^) colonies that contained the plasmid bacmid GalK gene replacing the pIX gene of the MAV-1 bacmid were identified. One of these colonies (pMAV-1ΔpIX::galK-C) was then used for the replacement substitution step, in which the pIX-eGFP fusion replaced the GalK gene.

To obtain the DNA fragment for the replacement substitution step, pMXD-FGFPpIX was digested with HpaI and PvuI, and the fragment corresponding to MAV-1 nt 2059 to 3700 (with the insertion of FLAG-eGFP at MAV-1 nt 3172) was gel purified. This fragment was introduced into pMAV-1ΔpIX::galK-C by recombineering; selection against the GalK cassette (and thus replacement by the pIX-eGFP gene) was achieved by selecting for resistance to 2-deoxy-galactose. Candidate colonies were identified by BamHI digestion, BAC DNA was purified from two candidate colonies using a Marligen Biosciences PowerPrep HP Plasmid Purification kit, and relevant junctions were verified by DNA sequencing. The MAV-1 genome was released from the bacmid by PmeI digestion, phenol-chloroform extracted, ethanol precipitated, and resuspended in water. The DNA was transfected into CMT-93 cells using FuGENE HD transfection reagent (Roche). Transfected wells were passaged after 1 week when no cytopathic effect (CPE) was visible. A second passage resulted in CPE, and GFP fluorescence was verified by immunofluorescence microscopy. Stocks were prepared from 3×-plaque-purified virus grown on mouse NIH 3T6 cells. The resulting MAV-1 strain with the pIX-eGFP is formally named MAV-1 inp903 and is referred to here descriptively as MAV-1.IXeGFP. MAV-1.IXeGFP stock titers are generally 10-fold lower than wt titers, but the virus has behaved like the wt strain in all assays performed to date (K. R. Spindler, unpublished data).

### Determination of MAV-1 loads by capture ELISA.

Whole brains were aseptically collected from euthanized mice. Brain homogenates were prepared as previously described (45) and assayed for MAV-1 load by capture ELISA (23). In each assay, an undiluted MAV-1 stock, PBS, and conditioned media were included as controls. The results of quantification of virus particles by capture ELISA correlate with infectious virus levels measured by plaque assay (45).

### Blood-brain barrier (BBB) permeability assay.

Mice were injected i.p. with 100 µl of 10% sodium fluorescein (Sigma)–DPBS 10 min prior to euthanasia. Mice were euthanized by CO_2_ asphyxiation. Mice were immediately perfused transcardially with 30 ml of ice-cold PBS. Brains were collected and snap-frozen until use. Sodium fluorescein levels in brain were determined as previously described (46) except that levels in the brain were not normalized to levels in serum. This is because in these MAV-1-susceptible SJL mice, moribund mice infected by both virus strains showed variable severe dehydration at the time of sodium fluorescein treatment, which affected total blood volume and thus serum concentrations. Fluorescence levels were measured on a Bio-Tek multidetection microplate reader with 485-nm excitation and 530-nm emission. Standards were used to calculate the sodium fluorescein content of brain.

### Measurement of TEER.

After initial isolation, pmBECs were plated onto collagen IV-coated 12-well transwells (Corning) (0.4-µm pore size). The medium was supplemented with 500 ng/ml hydrocortisone (Sigma catalog no. h6909) and 80% astrocyte-conditioned medium to aid in the formation of tight junctions. Astrocyte-conditioned medium was obtained from primary astroglia prepared as previously described (47). TEER was measured using an Endohm-12 electrical resistance apparatus (World Precision Instruments Inc.). TEER values (measured in ohms per square centimeter) were determined by subtraction of a blank well containing media alone. Cells were allowed to reach confluence over 7 to 10 days from the time of plating, until TEER levels reached 20 to 40 Ω·cm^2^. Cells were either mock infected or infected with wt MAV-1 or *pm*E109 virus at an MOI of 5. pmBEC growth medium with 2% fetal bovine serum and 2% newborn calf serum was used for the infection. TEER was measured on the day of infection and at 24-h intervals thereafter. Data are presented as percentages of the initial (day 0) TEER reading.

### Gelatin zymography.

Mouse brains that were mock infected or infected with wt or *pm*E109 viruses were homogenized in working buffer (50 mM Tris-HCl [pH 7.6], 150 mM NaCl, 5 mM CaCl_2_, 0.05% Brij 35, 0.02% NaN_3_) containing 1% Triton X-100 plus HALT protease inhibitor (catalog no. 78410; Thermo Scientific). Protein concentrations in the brain homogenates were measured by the use of a Pierce bicinchoninic acid (BCA) protein assay kit (catalog no. 23227; Thermo). All purifications were performed at 4°C. To concentrate the MMPs in brain lysates, gelatin-Sepharose beads (catalog no. 17095601; GE Healthcare) were washed twice and resuspended in working buffer. Brain homogenates (2 mg of protein) were mixed with 80 µl of gelatin-Sepharose beads and rotated for 2 h at 4°C. The beads were centrifuged at 16,200 × *g* for 5 min at 4°C. Pellets were washed with working buffer, and MMPs were eluted by resuspending the beads in 30 µl of elution buffer (1 × PBS and 10% dimethyl sulfoxide [DMSO]). Eluted supernatants were quantitated for MMPs by gelatin zymography. The eluted supernatants (15 µl) were mixed with nonreducing SDS gel sample buffer and electrophoresed on an 8% polyacrylamide gel (containing 1% SDS and 1 mg/ml gelatin) without boiling. After electrophoresis, the gels were rinsed with tap water and incubated in 2.5% Triton X-100 for 20 min twice. Finally, gels were washed in tap water and incubated in developing buffer (50 mM Tris-HCl [pH 7.6], 200 mM NaCl, 10 mM CaCl_2_, 0.02% Brij 35, 0.02% NaN_3_) for 72 h at 37°C. Proteins were stained by Coomassie brilliant blue R-250 solution (catalog no. B6529; Sigma). To visualize MMP activity, gels were destained with 10% acetic acid for 1 to 4 days. Band intensities in the gels were quantitated using Quantity one software and a Bio-Rad Gel Doc system.

### RT-qPCR.

RNA was isolated from whole brains using Trizol reagent (catalog no. 15596018; Life Technologies) or an RNeasy kit (catalog no. 74134; Qiagen) according to the manufacturers’ instructions. cDNA was synthesized from total RNA using random hexamers and murine leukemia virus reverse transcriptase (RT) (Invitrogen). Quantitative real-time PCR (qPCR) was performed using TaqMan PCR master mixes on an Applied Biosystems 7300 real-time PCR machine (Foster City, CA). TaqMan assays were used for claudin-5 (Mm00727012_s1; Applied Biosystems), occludin (Mm00500912_m1), zona occludens 2 (ZO-2; Mm00495620_m1), MMP2 (Mm00439498_m1), MMP3 (Mm00440295_m1), MMP9 (Mm00442991_m1), and glyceraldehyde-3-phosphate dehydrogenase (GAPDH) (4352339e) gene expression. cDNA corresponding to 50 to 100 ng of RNA equivalent was used in each reaction, and each sample was analyzed in triplicate. Quantitation was performed by normalizing target gene mRNA levels to GAPDH levels, and infected sample values are expressed relative to the mean of mock values, which was set to 1 for each gene. Undetermined values were assigned a *C_T_* value of 40. To calculate the statistical significance of between-group differences, we used Δ*C_T_* values, and for data presentation, we used 2^−ΔΔ*CT*^ values.

### Statistics.

Statistical analyses were performed with Prism 6 (GraphPad Software, Inc.). The specific tests used are indicated in the figure legends.
